# Supplementary material for: Analysis of Convergent Validity of Performance-Based Activities of Daily Living Assessed by PA-IADL Test in Relation to Traditional (Standard) Cognitive Assessment to Identify Older Adults with Mild Cognitive Impairment
Source: Behav Sci (Basel). 2023 Nov 27;13(12):975. doi: 10.3390/bs13120975 (PMC10740513; doi:10.3390/bs13120975)
Supplement: Supplementary file 1 [file behavsci-13-00975-s001.zip › behavsci-2679809-supplementary.pdf]

Supplementary Material

Section S1. Evaluation of the effectiveness of the pairing

Table S1. Sociodemographic and emotional state variables. Descriptive statistics in each Experimental Group [EG: Healthy (Controls) - MCI (Cases)], and inferential statistics of relationship with the variable EG.

Table S2. Statistics referring to the distribution, central tendency, variability, and position statistics in scale variables.

Graphic S1.G1. Graphical representation of the MMSE distribution in each EG

Section S2. Results of sub-objective 1.1

Table S3. Correlation among the 12 tasks of the PA-IADL test in the set of participants and correlation of age with the 12 tasks.

Table S4. Inferential results. MANCOVA Parallel slopes (Tasks T1, T2, T4, T5, T6, T8, T9, T10, and T11), ANCOVA NON-parallel slopes (Tasks T3 and T7) (in both, the grouping variable is EG and the covariate is the age]. ANOVA (Task T12).

Figure S1. Differential performance in the 12 tasks of the PA-IADL test in the groups of Cases (MCI) and Controls (Healthy) according to the age of the participants. Fit representation using iterative weighted least squares ([1]Loess *epanechnikov*).

Figure S2. In the upper part, the graph of the distribution of the Groups Centroids of the 6 groups resulting from the interaction between the levels of the variables EG and AR in the solution of the Discriminant analysis carried out in the training sample (selection of 70% Of the cases, approx, n = 50). At the bottom, the force of the discriminating function (introduction of variables by steps), through statistic F, in the corresponding samples to discriminate between the 6 groups resulting from the interaction (EG x AR).

Graphics S2.G1. Tasks in Row 1: T6, T8, T9 and T2. Left, *linear fit representation*. Right, iterative *weighted least squares* (*Loess epanechnikov*).

Graphics S2.G2. Tasks in Row 2: T12, T1, T10, T3, and T7. Left, *linear fit representation*. Right, iterative *weighted least squares* (*Loess epanechnikov*).

Graphics S2.G2 (continuation). Tasks in Row 2: T12, T1, T10, T3, and T7. Left, *linear fit representation*. Right, iterative *weighted least squares* (*Loess epanechnikov*).

Graphics S2.G3. Tasks in Row 3: T4, T5 and T11. Left, *linear fit representation*. Right, iterative *weighted least squares* (*Loess epanechnikov*).

Section S3. Results of sub-objective 1.2

Figure S3. Dendrogram of the hierarchical analysis solution using the furthest neighbor method and Ward's method in the training sample (selection of approx. 70% of the cases, n=42)

Figure S4. Dendrogram of the hierarchical analysis solution using the furthest neighbor method and Ward's method in the total sample of cases, N=64.

Figure S5. In the upper part, summary of the model in the classification of the participants using the two-stage cluster method. At the bottom, graph of the order of importance of the different tasks. The results in the solution of the training sample (selection of approx. 70% of the cases, n=50) and in the total sample, N=64, are presented on the left and right parts of the graph, respectively.

Figure S6. In the upper part, Histogram of the discriminant scores (Discriminant Analysis). The cut-off point of the classification is shown. At the bottom, Histogram of distances from the center of the cluster (K-means). The results in the solution of the training sample (selection of approx. 70% of the cases, n=50) and in the total sample, N=64, are presented on the left and right parts of the graph, respectively.

Table S5. Classification table (or confusion matrix) made up of the crossing of categories between the classification of participants based on the PA-IADL tasks carried out by the different cluster analysis procedures (Hierarchical Cluster using the Ward and furthest neighbor methods, two-stage cluster, discriminant analysis (inclusion by steps and K means), and the classification carried out using the CgA tests (starting point), and Kappa coefficient. Results carried out with the total sample.

Table S6. Percentage of coincidence and Kappa coefficient between the five cluster analysis procedures examined, and the classification made using the cognitive tests (starting point) in the total sample (N=64).

Section S4. Model of explicit consent of the people who participate in the workshops of active and healthy aging.

Section S1

Table S1. Sociodemographic and emotional state variables. Descriptive statistics in each Experimental Group [EG: Healthy (Controls) - MCI (Cases)], and inferential statistics of relationship with the variable EG.

			[A]Descriptive stadistics	[B]Relationship with EG	Graphic representation
	**Variable**	**EG**	**[X: % (n)] / [M; SD; CV]**	**[Phi / V;*p*] / [F;p;η^2]**	
**SD**	**Gender**	**Healthy**	M: 18.8% (n=6)<br>F: 81.3% (n=26)	Phi= -.039; *p*=.756	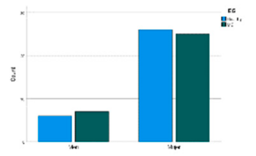
		**MCI**	M: 21.9% (n=7)<br>F:78.1% (n=25)		
	**M.St**	**Healthy**	**M: 40.6% (n=13)<br>S&D: 50% (n=16)<br>O: 9.4% (n=3)**	V= .135; *p*=.761	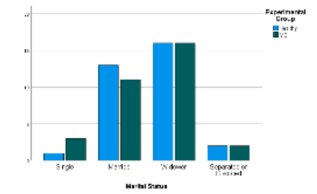
		**MCI**	**M: 34.4% (n=11)<br>S&D: 50% (n=16)<br>O: 15.7% (n=4)**		
	**Ys**	**Healthy**	M=6.66; SD=2.89; CV=.43	[C]F=.465; *p*=.489; η^2=.007	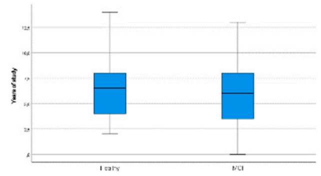
		**MCI**	M=6.13; SD=3.32; CV=.54		
**ES**	**GDS**	**Healthy**	[No D: 81.3% (n=26)]<br>[MD: 18.8% (n=6)]	Phi= .016; *p*=.901	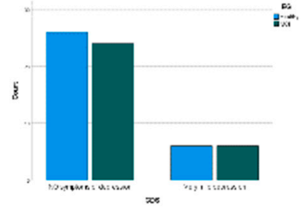
		**[D]MCI**	[No D: 80.30%(n=24)]<br>[MD: 20% (n=6)]		

Legend. V. SD=V. Sociodemographics; V.EE= Indicator variable of emotional state; M.St= Marital Status; Ys= Years spent studying; GDS= GDS=Geriatric Depression Screening Scale (Yesavage et al., 1982); EG= Experimental Group; [A]=[X: % (n)] / [M; SD; CV] descriptive statistics are shown according to the type of variable. If the variable is nominal, [X: % (n)], [variable level identifier: percentage (group size)], if the variable is quantitative, [M; SD; CV], [Mean; Standard deviation; Coefficient of Variation]. More statistics on quantitative variables are shown in the supplementary material. The variable levels are [EG: Cases or MCI; Controls or Healthy], [M.St: M= Married; S&D= Separated or Divorced; O= Others (due to their scarce representation, in order to analyze the relationship between M.St x EG, single persons and widows have been aggregated into a single group)], y [GDS: No D, and DL= No depression and mild depression]; [B]=[Phi / V; *p*] / [F; *p*; η^2] the relationship between two qualitative variables is examined by means of contingency tables (when the table is 2x2, the Phi coefficient is used and the relative risk (RR) is calculated. When the Table is other than 2x2, Kramer's V is used. The RR is [Gender: Healthy =.905; MCI=1.098], [GDS: Healthy =1.04 ; MCI=.960], and the relationship between a quantitative variable (Ys; years of study) and a classification variable, it is examined by Fisher's F (if there is heterogeneity of variances, the Brown-Forsythe F, F_{BF}, is used); *p*= probability of the outcome, assuming that Ho is true; η^2= partial square eta; [C]= the variances were homogeneous, df_1 (*df* numerator) and df_2 (*df* denominator) =1 and 60, respectively (herein after, respt.); [D]= two missing data.

Section S1

Table S2. Statistics referring to the distribution, central tendency, variability, and position statistics in scale variables

			Variable distribution measures			Measures of central tendency and variability[1]		Position measurements and variability[2]	
			**Mn-Mx (R)**	**$[\lambda_1; \lambda_2]$**	**S-W**	**M (SD)**	**CV**	**Me**	**$[P_{25}; P_{75}]$ (RI)**
**V. SD**	**Ys**	**Healthy**	2-14 (12)	[.657; .311]	.940 (*p*=.075)	6.66 (2.89)	.43	6.50	[4;8] (4)
		**MCI**	0-13 (13)	[-.161; -.676]	.952 (*p*=.162)	6.13 (3.32)	.54	6	[3.25;8] (5)
**V. FC**	**[C]MMSE**	**Healthy**	27-30 (3)	[-1.72; 2.29]	.653 (*p*=.000)	29.47 (.879)	.029	30	[29;30] (1)
		**MCI**	25-30 (5)	[-.953; -.358]	.792 (*p*=.000)	28.66 (1.63)	.056	29	[27;30] (3)
**CgA**	**CgT1**		1-8(7)	[.70;.45]	.935 (*p*=.059)	3,66 (1.79)	.48	4	[2;5] (3)
	**CgT 2**		13-39(26)	[,16; -.50]	.978 (*p*=.750)	24,31 (6.50)	.26	24	[19;30] (11)
	**CgT3**		10.5-59.2(48.7)	[1,52;2.98]	.875 (*p*=.002)	22,98 (10.65)	.46	20	[15,7;31] (15.2)
	**CgT4**	**Healthy**	1-13(12)	[,73;.56]	.953 (*p*=.187)	5,75 (2.82)	.49	5	[4;8] (4)
	**CgT5**		8-20(12)	[,39; -.61]	.958 (*p*=.262)	12,87 (3.25)	.25	13	[10;15] (5)
	**CgT6**		7-17(10)	[1,18;.45]	.842 (*p*=<.001)	9,94 (2.96)	.29	9	[8;11] (3)
	**CgT7**		2-13(11)	[,68; -.21]	.939 (*p*=.078)	6,47 (2.88)	.44	6	[4;8] (4)
	**CgT1**		0-7(6)	[,05; -1.09]	.912 (*p*=.033)	3,06 (1.93)	.63	4	[2;5] (3)
	**CgT 2**		5-35(30)	[,38; -.42]	.979 (*p*=.858)	17,97 (7.69)	.42	18	[14,5;26] (11.5)
	**CgT3**		2.25-54.75(52.5)	[2,31;7.41]	.810 (*p*=<.001)	14,28 (10.18)	.71	12	[9;19] (10)
	**CgT4**	**MCI**	0-10(10)	[,48; -.56]	.968 (*p*=.600)	4,13 (2.57)	.62	4	[2;6.5] (4.5)
	**CgT5**		7-16(9)	[-,14; -.69]	.964 (*p*=.493)	11,32 (2.44)	.21	11	[9,5;13] (3.5)
	**CgT6**		4-15(11)	[1,53; 3.58]	.843 (*p*=.001)	7,93 (2.20)	.27	8	[7;8.5] (1.5)
	**CgT7**		0-10(10)	[,35; -.30]	.956 (*p*=.573)	4,50 (2.41)	.53	4	[3;6.5] (3.5)

Legend. Mn-Mx (R)= Minimum value-Maximum value (Range); $[\lambda_1; \lambda_2]$= [Asymmetry; kurtosis]; K-S= Shapiro-Wilk; *p*= *p* value; M (SD)= Mean (Standard Deviation); CV= Coefficient of Variation; Me= Median; $[P_{25}; P_{75}]$ (RI)= [percentile values 25; percentile 75] (Interquartile range); For the rest, see Table S1. [CgT1=Word list I (first attempt) (WMS-III) ; CgT2= Word list I (four attempt) (WMS-III); CgT3= Symbols and digits test; CgT4= Word list II (WMS-III); CgT5= Digit span (WAIS-III); CgT6= Arithmetic (WAIS-III); CgT7=_Letter-number sequencing (WMS-III);

Section S1

Graphic S1.G1. Graphical representation of the MMSE distribution in each EG

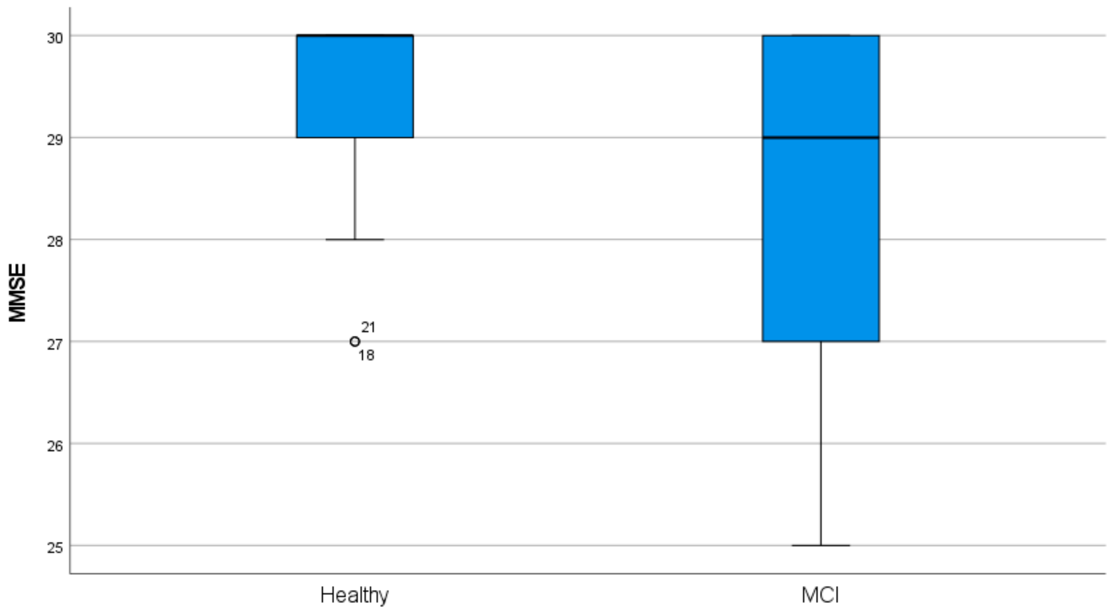

Legend. ◦=outlier.

Sección S2

Table S3. Correlation (r) among the 12 tasks of the PA-IADL test in the set of participants, and correlation of age with the 12 tasks.

	Age	T1	T2	T3	T4	T5	T6	T7	T8	T9	T10	T11	T12
Age	1	**-0.401****	**-0.348****	**-0.384****	**-0.573****	**-0.474****	**-0.431****	**-0.497****	**-0.531****	**-0.502****	**-0.379****	**-0.431****	-0.177
T1		1	0.416**	0.453**	0.414**	0.405**	0.569**	0.336**	0.408**	0.405**	0.653**	0.588**	0.553**
T2			1	0.263*	0.370**	0.235	0.221	0.333**	0.401**	0.343**	0.235	0.314*	0.209
T3				1	0.606**	0.525**	0.325**	0.412**	0.319*	0.284*	0.437**	0.446**	0.276*
T4					1	0.504**	0.384**	0.564**	0.558**	0.361**	0.376**	0.460**	0.336**
T5						1	0.172	0.383**	0.362**	0.336**	0.377**	0.483**	0.277*
T6							1	0.395**	0.425**	0.381**	0.429**	0.389**	0.251*
T7								1	0.363**	0.440**	0.410**	0.418**	0.189
T8									1	0.486**	0.360**	0.274*	0.337**
T9										1	0.325**	0.541**	0.172
T10											1	0.469**	0.438**
T11												1	0.335**
T12													1
Healthy	1	-0.317	-0.460**	-0.638**	-0.528**	-0.479**	-0.417*	-0.630**	-0.503**	-0.473**	-0.368*	-0.428*	-0.267
Age MCI	1	-0.531**	-0.276	-0.258	-0.746**	-0.556**	-0.450**	-0.406*	-0.575**	-0.550**	-0.468**	-0.510**	-0.181

Legend. T1=Fill in pill boxes; T2=Delayed recall of medical check; T3=Control over medication; T4=Event-based prospective memory (medical management); T5=Payment of workshop via bank; T6=Documentation Management; T7=Time-based prospective memory (administrative management); T8=Money management; T9=Management of bank documents; T10=Preparation of cooking recipe; T11=Bus route planning; T12=Recall/Recognition of ingredients; $r_{PA\text{-}IADL\text{-}AGE}$= Pearson correlation between PA-IADL Tasks and Age; Values of r ≥0 .20, r ≥ 0.50 and r ≥ 0.80 express a minimal, moderate and strong correlation, respect., (Cohen, 2013). * and **= the correlation is significant at the 0.05 and 0.01 level, respectively. Correlations greater than0 .50 are highlighted in red;

Cohen, J. *Statistical Power Analysis for the Behavioral Sciences.*; Routledge: London, UK, 2013. https://doi.org/10.4324/9780203771587.

Section S2.

Table S4. Inferential results. MANCOVA Parallel slopes (Tasks T1, T2, T4, T5, T6, T8, T9, T10, and T11), ANCOVA NON-parallel slopes (Tasks T3 and T7) (in both, the grouping variable is EG and the covariate is the age]. ANOVA (Task T12).

	**^{A}MANCOVA in the set of participants. Parallel slopes**						**ANCOVA in each age range. NON-parallel slopes**					**r $_{PA\text{-}IALD\text{-}AGE}$**	
**Tks**		**EG**	**Unadjusted M (SD)**	**Adjusted M (SD)**	**Tks**	**AR**			**Unadjusted M (SD)**	**Adjusted M (SD)**	**Tasks**	**Healthy**	**MCI**
**T1**	F=11.37; ***p*=0.000**; η^2=0.157	**Healthy**	4.06(1.39)	4.07	**T3**	**60-69**	F=10.67; ***p*=0.006**; η^2=0.433	**Healthy**	3.87(1.12)	3.95	T1	-0.317	**-0.531****
		**MCI**	2.97(1.51)	2.95				**MCI**	2.05(1.30)	1.98	T2	-0.460**	-0.276
**T2**	NS	**Healthy**	1.19(0.82)			**70-79**	F=18.30; ***p*=0.001**; η^2=0.604	**Healthy**	3.72(1.06)	3.75	T3	**-0.638****	-0.258
		**MCI**	0.88(0.94)					**MCI**	1.41(0.86)	1.37	T4	**-0.528****	**-0.746****
**T4**	F=15.22; ***p*=0.000**; η^2=0.200	**Healthy**	1.120(.87)	1.13		**>80**	NS	**Healthy**	2.06(1.03)		T5	-0.479**	**-0.556****
		**MCI**	0.53(0.68)	0.52				**MCI**	1.47(1.13)		T6	-0.417*	-0.450**
**T5**	F=15.42; ***p*=0.000**; η^2=0.202	**Healthy**	3.32(0.73)	3.33	**T7**	**60-69**	NS	**Healthy**	1.25(0.75)		T7	**-0.630****	-0.406*
		**MCI**	2.50(1.20)	2.49				**MCI**	0.77(0.75)		T8	**-0.503****	**-0.575****
**T6**	NS	**Healthy**	3.53(0.80)			**70-79**	F=5.88; ***p*=0.032**; η^2=0.329	**Healthy**	0.94(0.88)	1	T9	-0.473**	**-0.550****
		**MCI**	3.380(.83)					**MCI**	0.16(0.40)	0.07	T10	-0.368*	-0.468**
**T8**	NS	**Healthy**	1.34(0.65)			**>80**	NS	**Healthy**	0.13(.35)		T11	-0.428*	**-0.510****
		**MCI**	1.06(0.80)					**MCI**	0.11(.28)		T12	-0.267	-0.181
**T9**	NS	**Healthy**	2.34(1.28)										
		**MCI**	2(1.16)										
**T10**	F=14.03; ***p*=0.000**; η^2=0.187	**Healthy**	5.42(2.15)	5.43									
		**MCI**	3.60(2.13)	3.59									
**T11**	F=13.75**; *p*=0.000**; η^2=0.184	**Healthy**	6.25(1.66)	6.26									
		**MCI**	4.34(2.88)	4.32									
	**ANOVA in the set of participants**												
**T12**	F=20.60; ***p*=0.000**; η^2=0.252	**Healthy**	10.15(1.11)										
		**MCI**	8.59(1.64)										

Legend. Tks= Tasks; ^{A}= The result of the univariate tests provided by the SPSS MANCOVA output is shown; F= *Fisher's F*-test; η^2= the partial η^2; *p*= *p*-value; M (SD)= Mean (standard deviation); EG=Experimental group Healthy (Controls) and MCI (Cases); AR= age range 60-60, 70-79 y >80. Sample size in age range 60-60, 70-79 y >80, in Healthy n=8, 9 y 15, respt. and in MCI, n=9, 6 y 17 respt. T1=Fill in pill boxes; T2=Delayed recall of medical check; T3=Control over medication; T4=Event-based prospective memory (medical management); T5=Payment of workshop via bank; T6=Documentation Management; T7=Time-based prospective memory (administrative management); T8=Money management; T9=Management of bank documents; T10=Preparation of cooking récipe; T11=Bus route planning; T12=Recall/Recognition of ingredients; r $_{PA\text{-}IALD\text{-}AGE}$= Pearson correlation between PA-IALD tasks and age; * and **= the correlation is significant at the 0.05 and 0.01 level, respectively.

Sección S2

Figure S1. Differential performance in the 12 Tasks of the PA-IADL in the groups of Cases (MCI) and Controls (Healthy) according to the age of the participants.. Fit representation using iterative weighted least squares ([1]Loess *epanechnikov*).

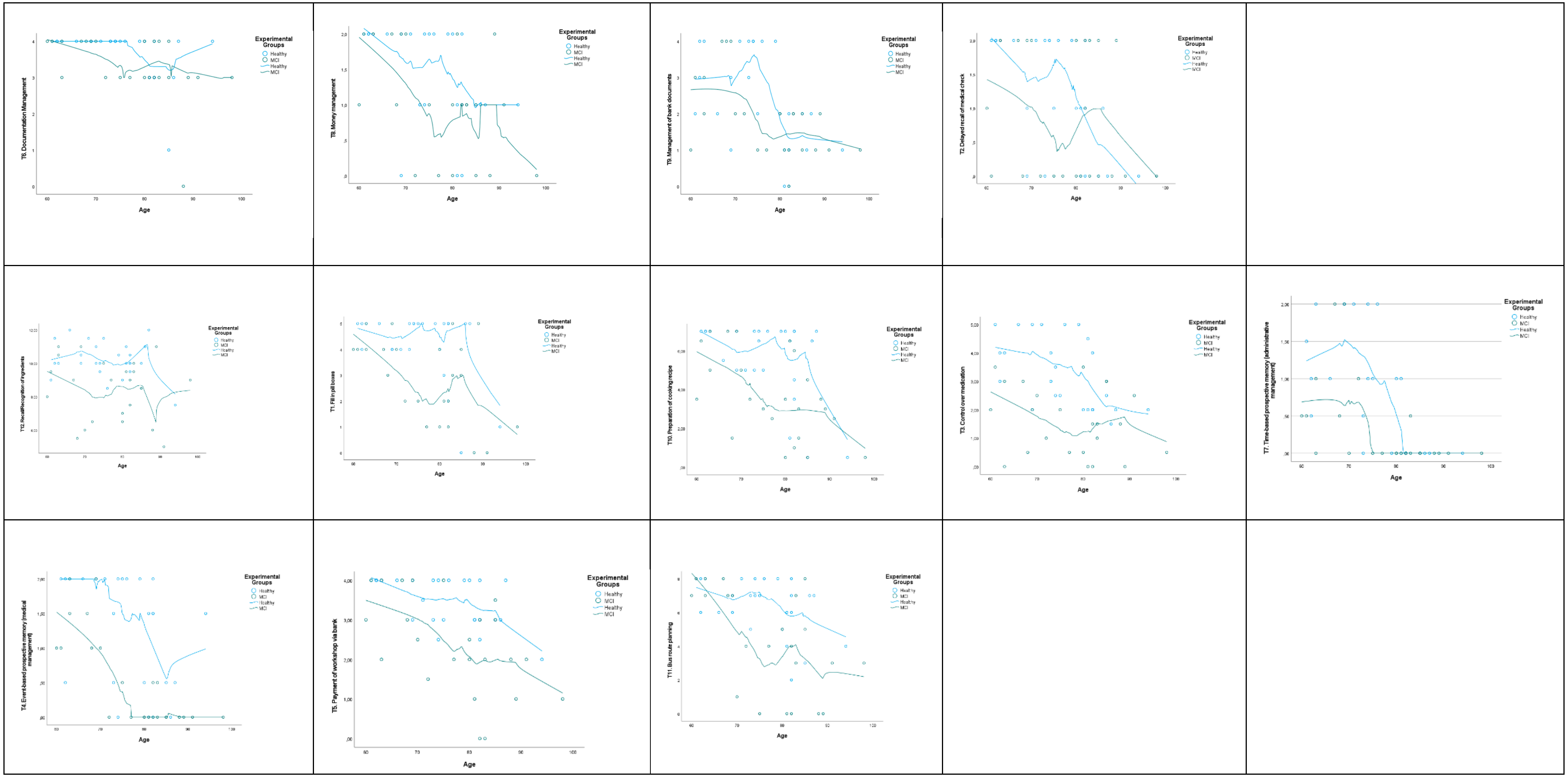

Legend. The graphics are placed in order of shape. Tasks in Row 1: T6, T8, T9, and T2; Tasks in Row 2: T12, T1, T10, T3, and T7; Tasks in Row 3: T4, T5 and T11; [1]= data located close to each particular point receives greater weight than extreme data.

Sección S2. Graphics S2.G1. Tasks in Row 1: T6, T8, T9 and T2. Left, *linear fit representation*. Right, iterative *weighted least squares* (*Loess epanechnikov*).

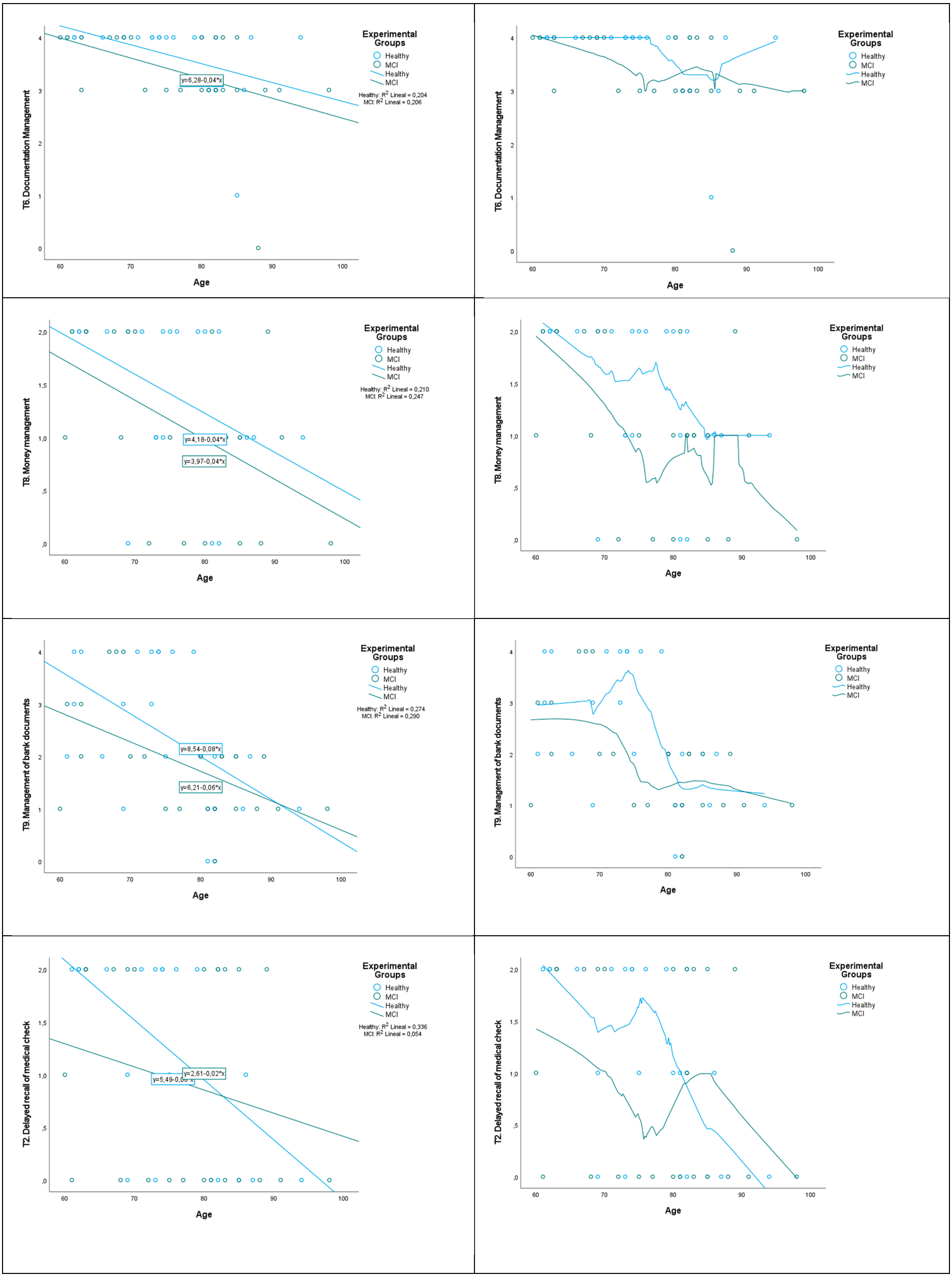

Sección S2. Graphics S2.G2. Tasks in Row 2: T12, T1, T10, T3, and T7. Left, *linear fit representation*. Right, iterative *weighted least squares* (*Loess epanechnikov*).

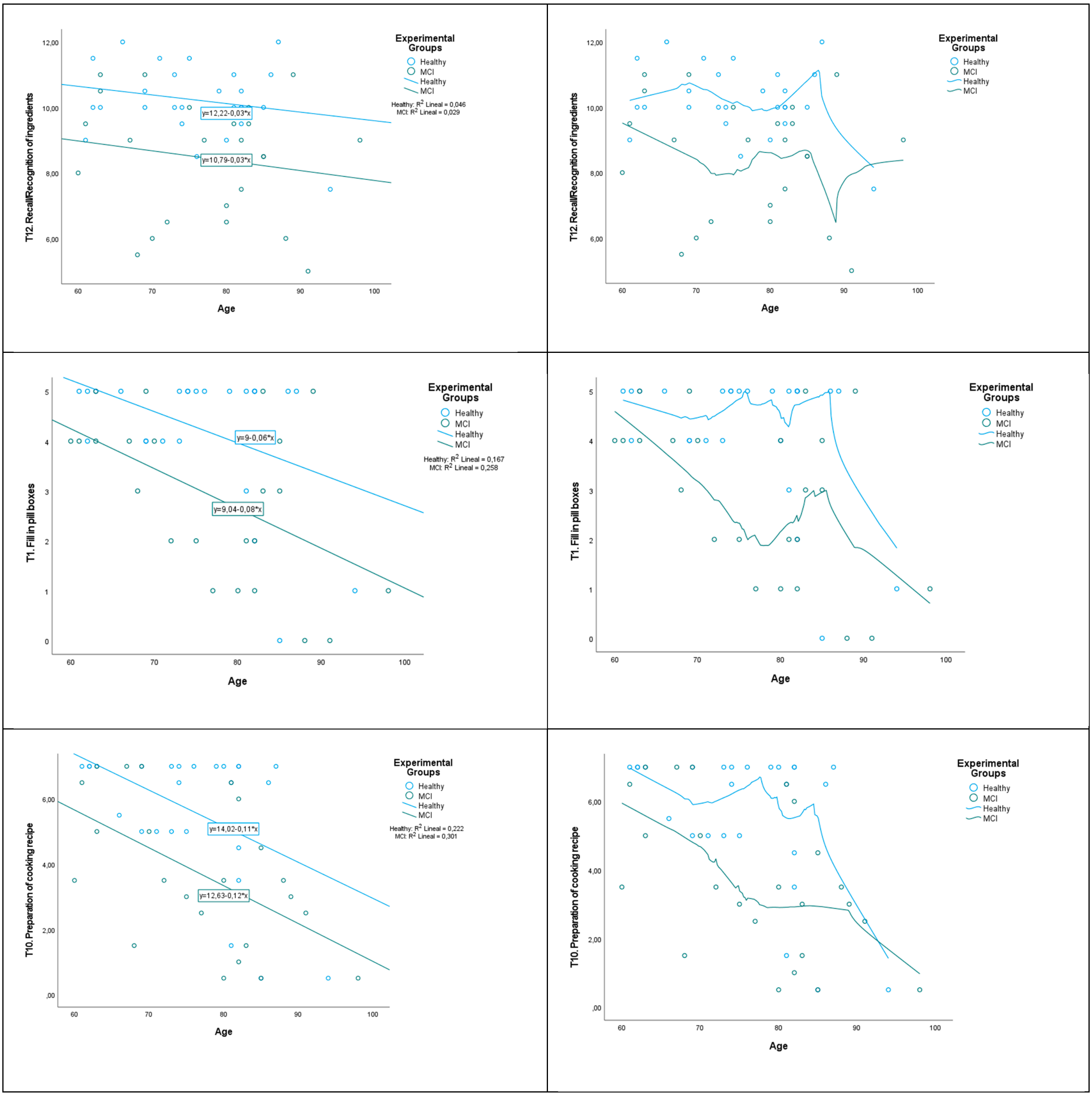

Sección S2. Graphics S2.G2 (**continuation**). Tasks in Row 2: T12, T1, T10, T3, and T7. Left, *linear fit representation*. Right, iterative *weighted least squares* (*Loess epanechnikov*).

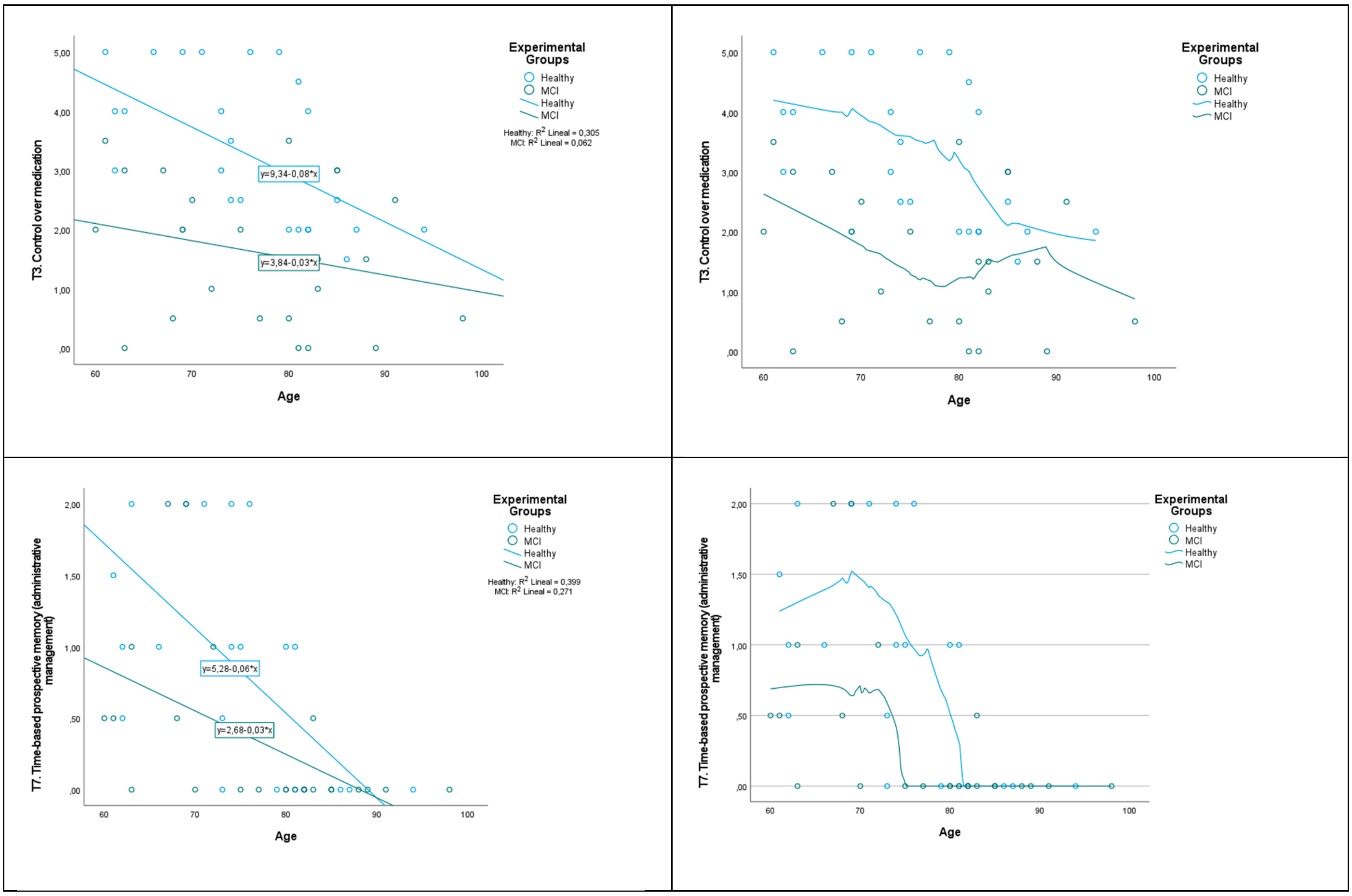

Sección S2. Graphics S2.G3. Tasks in Row 3: T4, T5 and T11. Left, *linear fit representation*. Right, iterative *weighted least squares* (*Loess epanechnikov*).

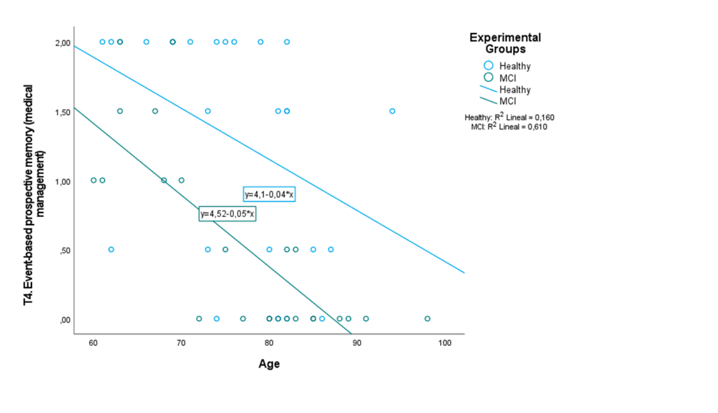

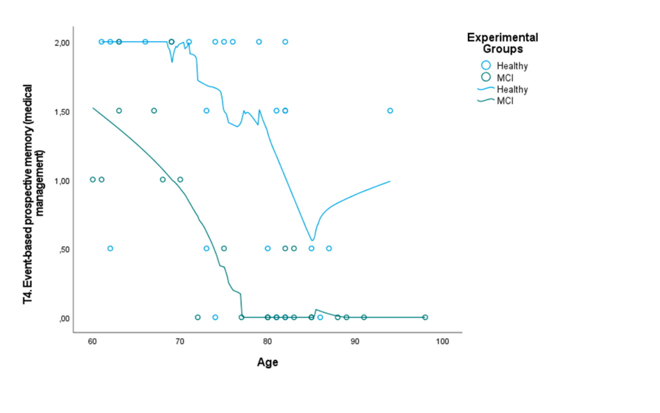

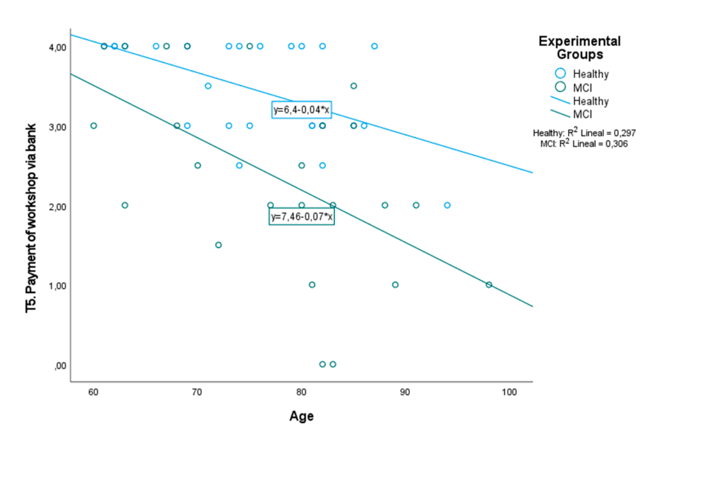

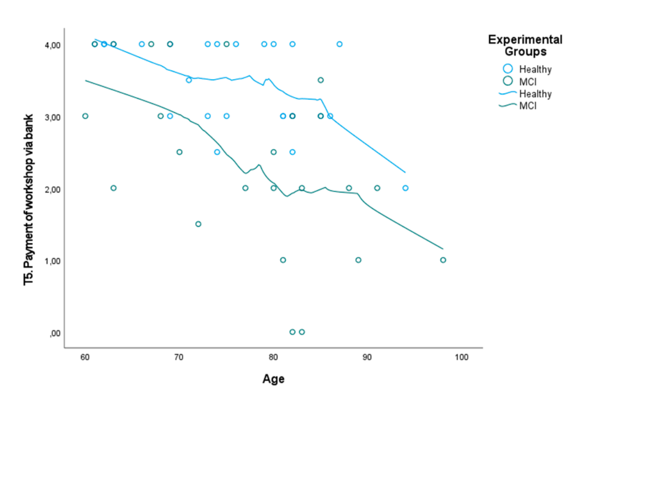

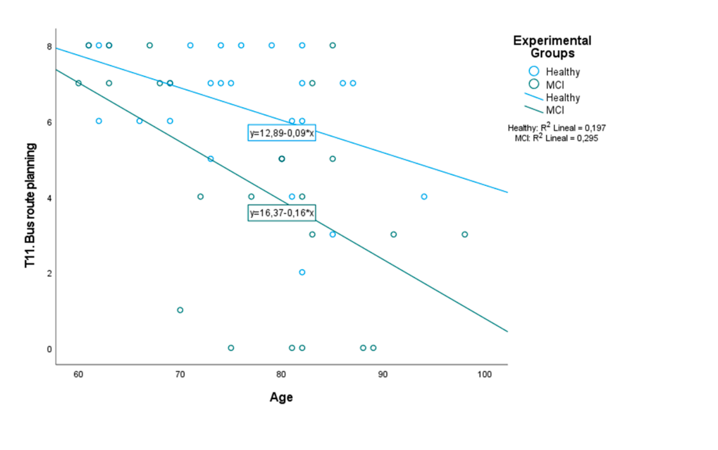

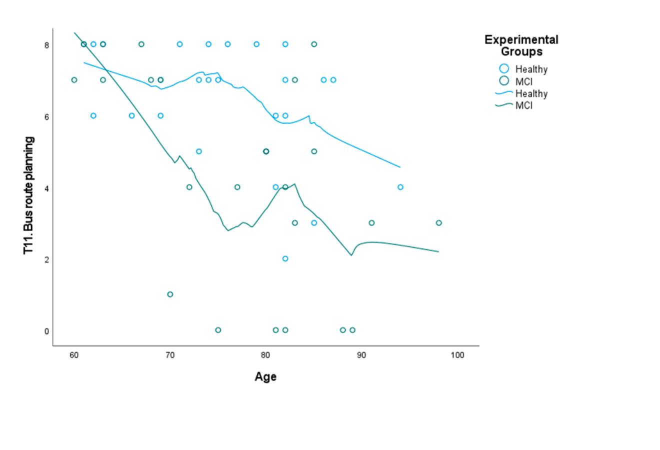

Sección S2

Figure S2. In the upper part, the graph of the distribution of the Groups Centroids of the 6 groups resulting from the interaction between the levels of the variables EG and AR in the solution of the Discriminant analysis carried out in the training sample (selection of 70% Of the cases, approx, n = 50). At the bottom, the force of the discriminating function (introduction of variables by steps), through statistic F, in the corresponding samples to discriminate between the 6 groups resulting from the interaction (EG x AR).

Training sample n=50. Cc=70%

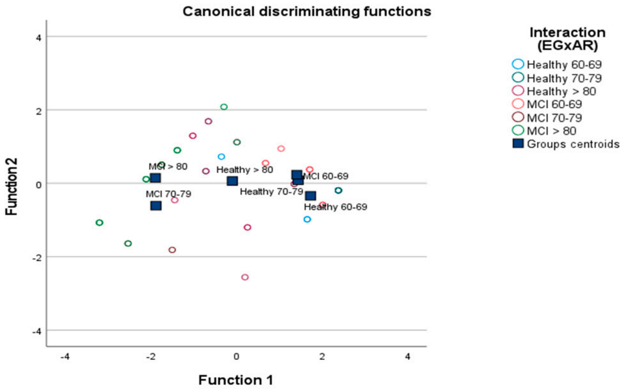

	Training sample. [A]Step 1 : T4					
	**H 60-69**	**H 70-79**	**H >80**	**MCI 60-69**	**MCI 70-79**	**MCI> 80**
**H 60-69**		0.9380(.338)	11.192(0.002)	1.374(0.247)	15.588(0.000)	40.8830(.000)
**H 70-79**			5.772(0.021)	0.059(0.810)	10.385(0.002)	30.862(0.000)
**H >80**				4.201(0.046)	2.155(0.149)	11.348(0.002)
**MCI 60-69**					8.694(0.005)	25.579(0.000)
**MCI 70-79**						0.836(0.365)

	Training sample. [B]Step 2: T11					
	**H 60-69**	**H 70-79**	**H >80**	**MCI 60-69**	**MCI 70-79**	**MCI> 80**
**H 60-69**		0.482(0.621)	7.331(0.002)	0.742(0.482)	16.344(0.000)	29.811(0.000)
**H 70-79**			5.351(0.008)	0.044(0.957)	15.017(0.000)	26.970(0.000)
**H >80**				4.808(0.013)	5.219(0.009)	9.457(0.000)
**MCI 60-69**					14.372(0.000)	24.334(0.000)
**MCI 70-79**						0.854(.0433)

Legend. [A, B, C, D]= degrees of freedom in steps 1 and 2 of the statistic F [1:44 and 2:43, respt.]; H=Healthy

Section S3

Figure S3. Dendrogram of the hierarchical analysis solution using the furthest neighbor method and Ward's method in the training sample (selection of approx. 70% of the cases, n=42)

Furthest neighbor n=42	Ward´s method n=42

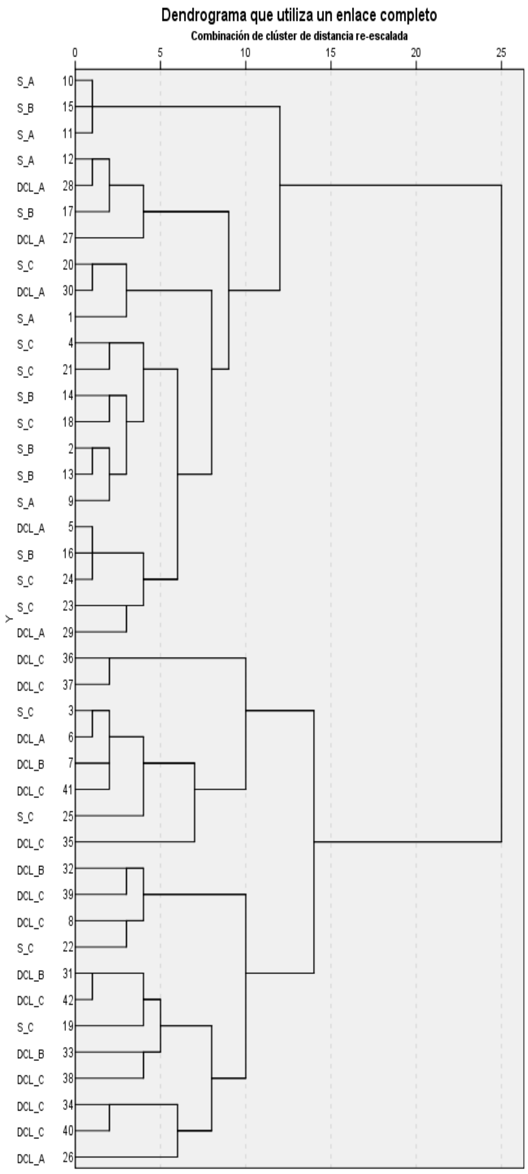

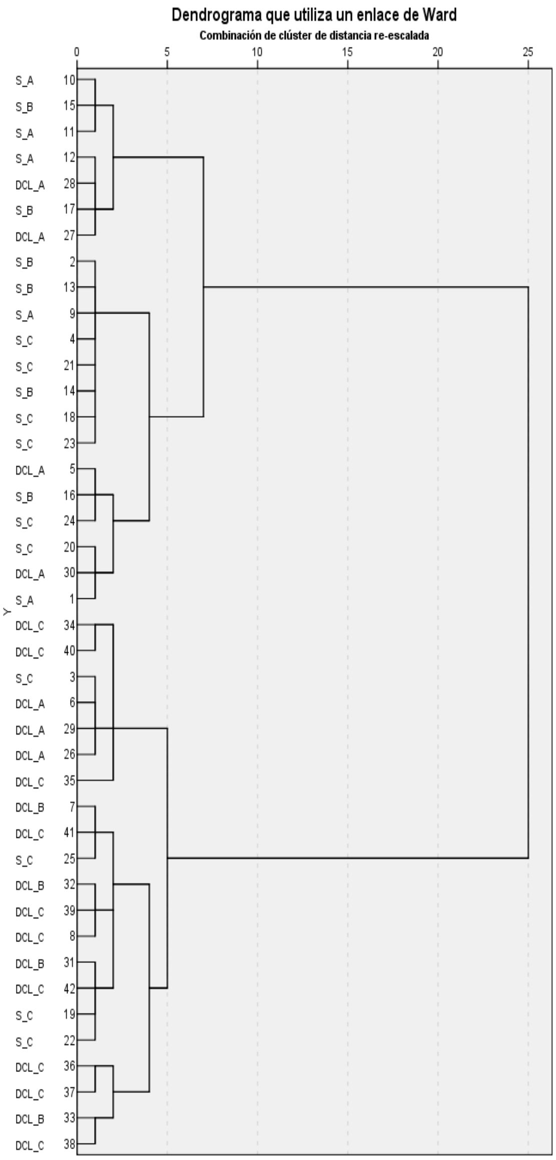

Legend. S, MCI= Healthy participants (Controls) and MCI (cases) classified by cognitive tests; A, B and C= indicates age of 60-69 years, 70-79 years and over 80 years respectively. The letter within the graph appears in Spanish because the SPSS program from which the data have been analyzed was installed in Spanish, and SPSS does not allow these graphs to be modified. If the reader has any questions, please get in touch with the corresponding author, and we will resolve your doubts.

Section S3

Figure S4. Dendrogram of the hierarchical analysis solution using the furthest neighbor method and Ward's method in the total sample of cases, N=64.

Furthest neighbor N=64	Ward´s method N=64
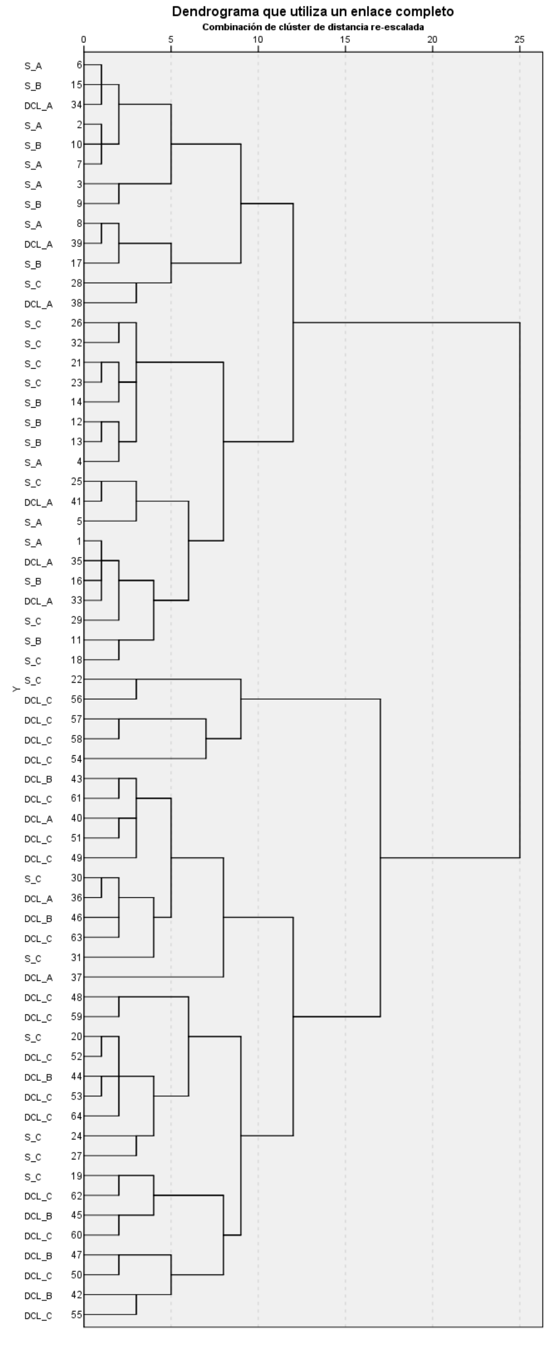	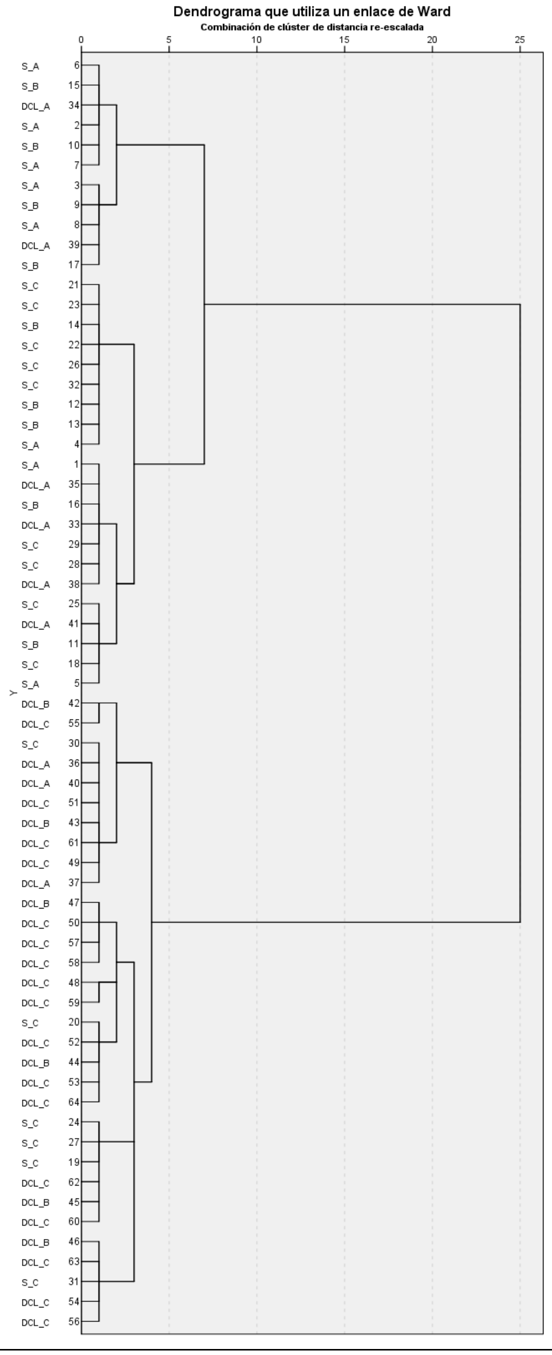

Legend. See Figure S3.

Section S3

Figure S5. In the upper part, summary of the model in the classification of the participants using the two-stage cluster method. At the bottom, graph of the order of importance of the different tasks. The results in the solution of the training sample (selection of approx. 70% of the cases, n=50) and in the total sample, N=64, are presented on the left and right parts of the graph, respectively.

Sample training n=50. Cc=70%	Total sample N=64.
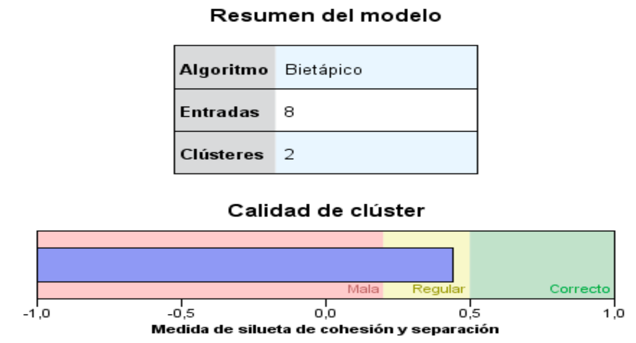	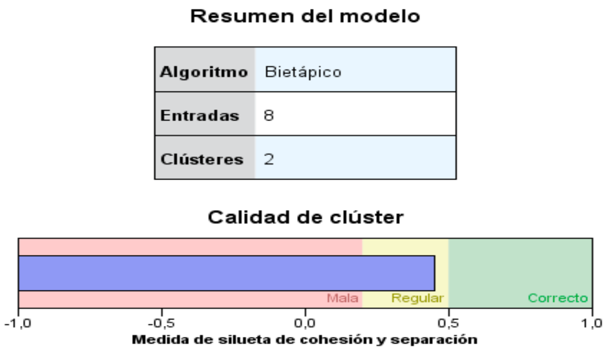
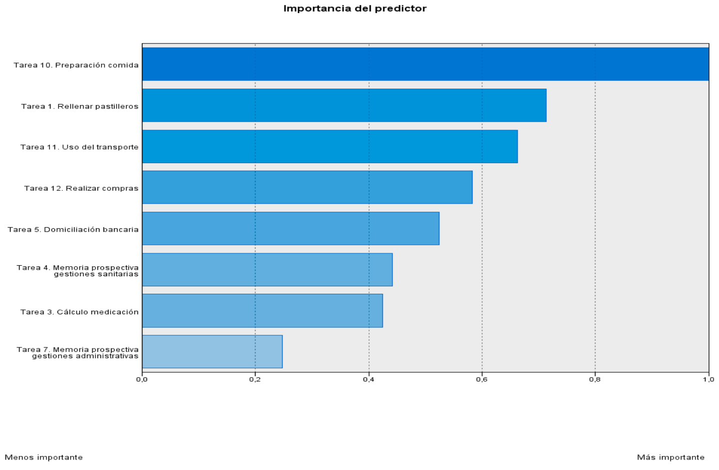	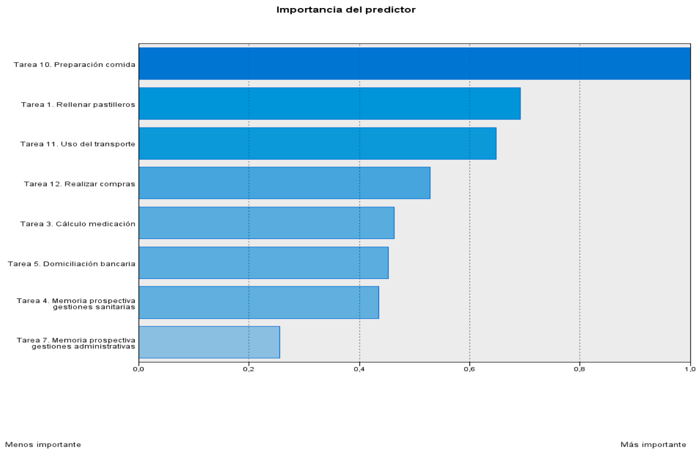

Legend. The letter within the graph appears in Spanish because the SPSS program from which the data have been analyzed was installed in Spanish, and SPSS does not allow these graphs to be modified. If the reader has any questions, please get in touch with the corresponding author, and we will resolve your doubts.

Section S3

Figure S6. In the upper part, Histogram of the discriminant scores (Discriminant Analysis). The cut-off point of the classification is shown. At the bottom, Histogram of distances from the center of the cluster (K-means). The results in the solution of the training sample (selection of approx. 70% of the cases, n=50) and in the total sample, N=64, are presented on the left and right parts of the graph, respectively.

Sample training n=50. Cc=70%	Total sample N=64.
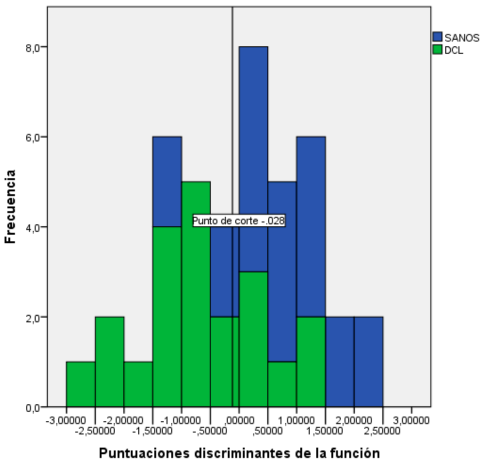	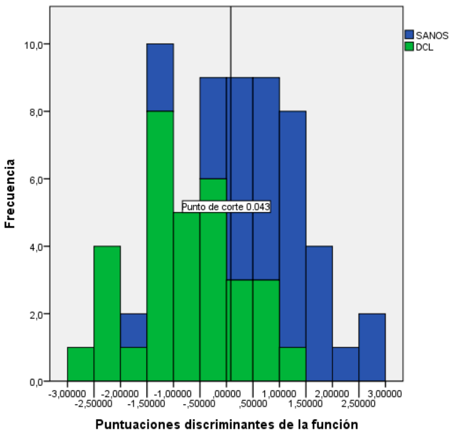
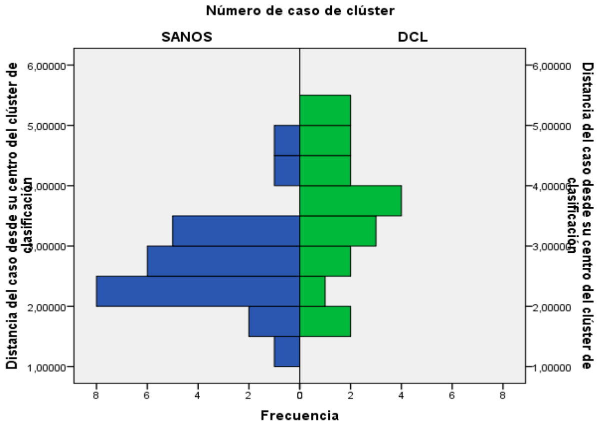	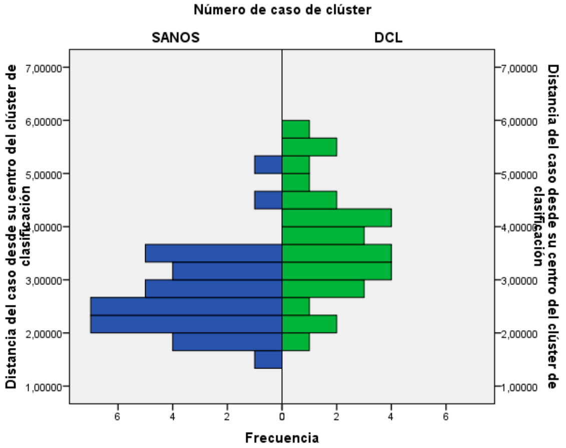

Legend. We represent it differently because the discriminant analysis gives scores of the discriminant functions that are positive and negative values, the K means procedure gives distances from the center of the group, therefore all are positive. What we see in both graphs is the distribution of both values.

Section S3

Table S5. Classification table (or confusion matrix) made up of the crossing of categories between the classification of participants based on the PA-IADL tasks carried out by the different cluster analysis procedures (Hierarchical Cluster using the Ward and furthest neighbor methods, two-stage cluster, discriminant analysis (inclusion by steps and K means), and the classification carried out using the CgA tests (starting point), and Kappa coefficient.Results carried out with the total sample.

**Classification method**		**M. Clúster $J_{W.M}$**			[A]**Kappa**
		**Controls**	**Cases**	**Total F** Cl.CgA	
					**M. Clúster-Cl.CgA**
Cl.CgA	**Healthy**	26	6	**32**	**0.625 (*p*=0.000)**
	**MCI**	6	26	**32**	
	**Total C**	32	32	**64**	
	**%Cc**	**81.24%**			
		**M. Cluster** $J_{FN.M}$			
		**Controles**	**Casos**		
Cl.CgA	**Healthy**	25	7	**32**	**0.594 (*p*=0.000)**
	**MCI**	6	26	**32**	
	[B]**Total C**	31	33	64	
	**%Cc**	**79.68%**			
		**M. Cluster Two-Stage**			
		**Controles**	**Casos**		
Cl.CgA	**Healthy**	26	6	**32**	**0.594 (*p*=0.000)**
	**MCI**	7	25	**32**	
	**Total C**	33	31	64	
	**%Cc**	79.68%			
		**M. Cluster Discriminant**			
		**Controls**	**Cases**		
Cl.CgA	**Healthy**	26	6	**32**	**0.594 (*p*=0.000)**
	**MCI**	7	25	**32**	
	**Total C**	33	31	64	
	**%Cc**	79.68%			
		**M. Cluster K-Means**			
		**Controls**	**Cases**		
Cl.CgA	**Healthy**	26	6	**32**	**0.531 (*p*=0.000)**
	**MCI**	9	23	**32**	
	**Total C**	35	29	64	
	**%Cc**	**76.56%**			

Legend. %Cc=percentage of coincidence in the classification; Cl.CgA=classification using cognitive tests; M.Cluster= Classification carried out by the different exploratory classification procedures; JW.M, JFN.M = Classification carried out by the Hierarchical Cluster procedure using the Ward method and the farthest neighbor method, respectively; A=The Kappa coefficient is made between the classification made by each of the classification methods used in the total sample and the classification made by cognitive tests (M. Cluster-Cl.CgA)

Section S3

Table S6. Percentage of coincidence and Kappa coefficient between the five cluster analysis procedures examined, and the classification made using the cognitive tests (starting point) in the total sample (N=64).

**Classification methods**			**M.Cluster**									
	**Cl.Cg**		**$J_{W.M}$**		**$J_{FN.M}$**		**Two-Stage**		**Discriminant**		**K-Means**	
	[1]%Mc	[2]Kappa	%Mc	Kappa	%Mc	Kappa	%Mc	Kappa	%Mc	Kappa	%Mc	Kappa
**$J_{W.M}$**	81.24 (12)	0.625 (*p*=0.000)	-----	-----	98.43 (1)	0.969 (*p*=0.000)	96.87 (2)	0.938 (*p*=0.000)	89.06 (7)	0.781 (*p*=0.000)	93.75 (4)	0.87 (*p*=0.000)
**$J_{FN.M}$**	79.68 (13)	0.594 (*p*=0.000)			-----	-----	98.43 (1)	0.969 (*p*=0.000)	87.5 (8)	0.750 (*p*=0.000)	95.31 (3)	0.90 (*p*=0.000)
**Two-Stage**	79.68 (13)	0.594 (*p*=0.000)					-----	-----	87.5 (8)	0.750 (*p*=0.000)	96.87 (2)	0.938 (*p*=0.000)
**Discriminant**	79.68 (13)	0.594 (*p*=0.000)							-----	-----	84.37 (10)	0.687 (*p*=0.000)
**K-Means**	76.56 (15)	0.531 (*p*=0.000)									-----	-----

Legend. M= Method; $J_{W.M}$, $J_{FN.M}$ = Classification carried out by the Hierarchical Cluster procedure using the Ward method and the farthest neighbor method, respectively; Cl.Cg= classification performed using cognitive tests (traditional classification mode. Starting point); %Mc= percentage of matching cases in the classification; [1]= the number of mismatched cases is shown in parentheses; *p*= *p* value.

Section S4

EXPLICIT CONSENT OF THE PEOPLE WHO PARTICIPATE IN THE ACTIVE AND HEALTHY AGING WORKSHOPS

NAVA, on date.........................

MANCOMUNIDAD COMARCA DE LA SIDRA is **Responsible for the processing** of the personal data of the Data Subject and informs you that this data will be processed in accordance with the provisions of Regulation (EU) 2016/679, of 27 April (RGPD), and Organic Law 3/2018, of 5 December (LOPDGDD), and therefore provides you with the following processing information:

Purposes and legitimacy of the processing: Research purposes, to evaluate cognitive capacity and everyday cognition through different neuropsychological tests that will allow data to be collected over time to analyze the evolution of these capacities in people who participate in the active and healthy ageing workshops, based on the consent of the data subject, art. 6.1.a RGPD.

Data retention criteria: data shall be retained for no longer than is necessary to maintain the purpose of the processing or as long as there are legal requirements for their safekeeping and when no longer necessary for that purpose, they shall be erased with appropriate security measures to ensure the anonymisation of the data or their complete destruction.

Data communication: The data collected will be treated anonymously for the purpose of carrying out different statistical analyzes and may be communicated to the interested parties (if they request it) or communicated in meetings or scientific publications, however, the identity or data that can identify the participants will never be provided and confidentiality will be always maintained.

Rights of the Interested Party: -

- Right to withdraw consent at any time.
- Right of access, rectification, portability and deletion of your data, and limitation or opposition to its processing.
- Right to file a claim with the Control Authority (www.aepd.es) if you consider that the treatment does not comply with current regulations.

Contact details for exercising your rights:

MANCOMUNIDAD COMARCA DE LA SIDRA. PARAES, 47 - 33529 NAVA (Asturias). E-mail: dpd@lacomarcadelasidra.com

The Data Subject or his or her legal representative consents to the processing of his or her data in the terms set out above:

Name ...., with NIF............................

Legal representative of: ...., with NIF ....

Signature:

CONSENTIMIENTO EXPLÍCITO DE LAS PERSONAS QUE PARTICIPAN EN LOS TALLERES DE ENVEJECIMIENTO ACTIVO Y SALUDABLE

NAVA, en fecha ....

MANCOMUNIDAD COMARCA DE LA SIDRA es el **Responsable del tratamiento** de los datos personales del **Interesado** y le informa de que estos datos se tratarán de conformidad con lo dispuesto en el Reglamento (UE) 2016/679, de 27 de abril (RGPD), y la Ley Orgánica 3/2018, de 5 de diciembre (LOPDGDD), por lo que se le facilita la siguiente información del tratamiento:

Fines y legitimación del tratamiento: Fines de investigación, evaluar la capacidad cognitiva y la cognición cotidiana a través de diferentes pruebas neuropsicológicas que permitirá recopilar datos a lo largo del tiempo con el objeto de analizar la evolución de dichas capacidades en personas queparticipan en los talleres de envejecimiento activo y saludable, en base al consentimiento del interesado, art. 6.1.a RGPD.

Criterios de conservación de los datos: se conservarán durante no más tiempo del necesario para mantener el fin del tratamiento o mientras existan prescripciones legales que dictaminen su custodia y cuando ya no sea necesario para ello, se suprimirán con medidas de seguridad adecuadas para garantizar la anonimización de los datos o la destrucción total de los mismos.

Comunicación de los datos: Los datos recogidos serán tratados de forma anónima con el fin de llevara cabo diferentes análisis estadísticos y podrán ser reportados a los interesados (si así lo solicitan) o comunicados en reuniones científicas o publicaciones, sin embargo, nunca será facilitada la identidad odatos que puedan identificar a los participantes, manteniéndose en todo momento la confidencialidad.

Derechos que asisten al Interesado:

- Derecho a retirar el consentimiento en cualquier momento.
- Derecho de acceso, rectificación, portabilidad y supresión de sus datos, y de limitación u oposición a su tratamiento.
- Derecho a presentar una reclamación ante la Autoridad de control (www.aepd.es) si considera que el tratamiento no se ajusta a la normativa vigente.

Datos de contacto para ejercer sus derechos:

MANCOMUNIDAD COMARCA DE LA SIDRA. PARAES, 47 - 33529 NAVA (Asturias). E-mail: dpd@lacomarcadelasidra.com

El **Interesado** o su representante legal consiente el tratamiento de sus datos en los términos expuestos:

Nombre ...., con NIF ....

Representante legal de ...., con NIF ....

Firma:
